# (Re)building the nervous system: A review of neuron–glia interactions from development to disease

**DOI:** 10.1111/jnc.16258

**Published:** 2024-12-16

**Authors:** Matthew D. Demmings, Luana da Silva Chagas, Marianela E. Traetta, Rui S. Rodrigues, Maria Florencia Acutain, Evgeny Barykin, Ashok Kumar Datusalia, Liliana German‐Castelan, Vanesa S. Mattera, Pedzisai Mazengenya, Cecilia Skoug, Hisashi Umemori

**Affiliations:** ^1^ Neuroscience Program, Schulich School of Medicine and Dentistry University of Western Ontario London Ontario Canada; ^2^ Department of Neurobiology and Program of Neurosciences, Institute of Biology Fluminense Federal University Niterói Rio de Janeiro Brazil; ^3^ Instituto de Biología Celular y Neurociencia (IBCN), Facultad de Medicina Conicet Buenos Aires Argentina; ^4^ University of Bordeaux, INSERM, Neurocentre Magendie U1215 Bordeaux France; ^5^ Engelhardt Institute of Molecular Biology Russian Academy of Sciences Moscow Russia; ^6^ Department of Pharmacology and Toxicology National Institute of Pharmaceutical Education and Research (NIPER Raebareli) Raebareli UP India; ^7^ Instituto de Química y Fisicoquímica Biológica (IQUIFIB—FFyB‐UBA) Universidad de Buenos Aires Buenos Aires Argentina; ^8^ Center of Medical and bio‐Allied Health Sciences Research, College of Medicine Ajman University Ajman United Arab Emirates; ^9^ Department of Neuroscience, Physiology & Pharmacology, Centre for Cardiovascular and Metabolic Neuroscience University College London London UK; ^10^ Department of Neurology, F.M. Kirby Neurobiology Center Boston Children's Hospital, Harvard Medical School Boston Massachusetts USA

**Keywords:** astrocyte, brain development and function, glial dysfunction, microglia, neurodegenerative and neuropsychiatric disorders, oligodendrocyte

## Abstract

Neuron–glia interactions are fundamental to the development and function of the nervous system. During development, glia, including astrocytes, microglia, and oligodendrocytes, influence neuronal differentiation and migration, synapse formation and refinement, and myelination. In the mature brain, glia are crucial for maintaining neural homeostasis, modulating synaptic activity, and supporting metabolic functions. Neurons, inherently vulnerable to various stressors, rely on glia for protection and repair. However, glia, in their reactive state, can also promote neuronal damage, which contributes to neurodegenerative and neuropsychiatric diseases. Understanding the dual role of glia—as both protectors and potential aggressors—sheds light on their complex contributions to disease etiology and pathology. By appropriately modulating glial activity, it may be possible to mitigate neurodegeneration and restore neuronal function. In this review, which originated from the International Society for Neurochemistry (ISN) Advanced School in 2019 held in Montreal, Canada, we first describe the critical importance of glia in the development and maintenance of a healthy nervous system as well as their contributions to neuronal damage and neurological disorders. We then discuss potential strategies to modulate glial activity during disease to protect and promote a properly functioning nervous system. We propose that targeting glial cells presents a promising therapeutic avenue for rebuilding the nervous system.

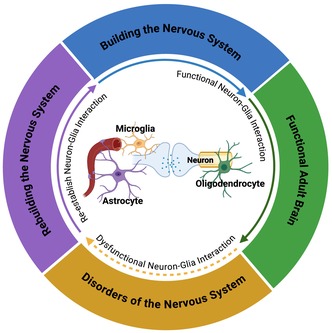

## Graphical abstract

Neuron–glia interactions play a vital role in brain development, influencing processes like neuronal differentiation, synapse development, and myelination. In the mature brain, glial cells maintain homeostasis, support synaptic function/plasticity, and protect against damage. However, when neuron–glia interactions are disrupted, activated/reactive glia can lead to neuronal damage, contributing to neurodegenerative and neuropsychiatric disorders. Given their dual role as both protectors and contributors to neuronal damage, modulating glial activity presents a promising therapeutic strategy to restore neural function and promote recovery in disorders of the nervous system.

**Abbreviations**
AAV: adeno‐associated virus
AD: Alzheimer's disease
ALS: amyotrophic lateral sclerosis
AMPA: α‐amino‐3‐hydroxy‐5‐methyl‐4‐isoxazolepropionic acid
APOE4: apolipoprotein E4
APP: amyloid precursor protein
ATG5: autophagy related 5
ATP: adenosine triphosphate
Aβ: amyloid β
BBB: blood–brain barrier
BDNF: brain‐derived neurotrophic factor
BLA: basolateral amygdala
C1q: complement component 1q
C3: complement component 3
C3a: complement component C3a
C3aR: C3a receptor
C4A: complement component C4a
C5: complement component 5
Ca2+: calcium ion
CBF: cerebral blood flow
CCL2: chemokine C‐C motif ligand 2
CNS: central nervous system
CNTF: ciliary neurotrophic factor
CNTFRα: ciliary neurotrophic factor receptor alpha
COX‐2: cyclooxygenase 2
CR3: complement receptor 3
CRP: C‐reactive protein
CSPGs: chondroitin sulfate proteoglycans
CX3CR1: C‐X3‐chemokine receptor 1
CXCL1: C‐X‐C motif chemokine ligand 1
CXCR4: C‐X‐C chemokine receptor 4
EAE: experimental autoimmune encephalomyelitis
ECs: endothelial cells
EGFR: epidermal growth factor receptor
FGF‐2: fibroblast growth factor‐2
GFAP: glial fibrillary acidic protein
GLP1: glucagon‐like peptide 1
GLP‐1R: glucagon‐like peptide‐1 receptor
GM‐CSF: granulocyte macrophage colony‐stimulating factor
Gpc4/6: glypincan 4 and 6
HD: Huntington's disease
HSPGs: heparan sulfate proteoglycans
IGF‐1: insulin‐like growth factor‐1
IL‐1: interleukin‐1
IL‐10: interleukin‐10
IL‐1β: interleukin‐1β
IL‐2: interleukin‐2
IL‐6: interleukin‐6
INF‐γ: interferon gamma
iPSCs: induced pluripotent stem cells
JAK: janus kinase
KSPGs: keratan sulfate proteoglycans
LAMs: leukocyte adhesion molecules
L‐DOPA: levodopa
LIF: leukemia inhibitory factor
LPS: lipopolysaccharide
LRRK2: leucine‐rich repeat kinase 2
LTD: long‐term depression
LTP: long‐term potentiation
MAG: myelin‐associated glycoprotein
MAO‐B: monoamine oxidase B
MBP: myelin basic protein
MDD: major depressive disorder
MEGF10: multiple EGF‐like domains 10
MERTK: mer tyrosine kinase
mGluR5: metabotropic glutamate receptor 5
MPTP: 1‐methyl‐4‐phenyl‐1,2,3,6‐tetrahydropyridine
MS: multiple sclerosis
mTOR: mechanistic target of rapamycin
NCAM: neural cell adhesion molecule
NG2: neuronal/glial antigen 2
NGF: nerve growth factor
NLRP3: NOD‐, LRR‐ and pyrin domain‐containing protein 3
NMDA: N‐methyl‐d‐aspartate
nNOS: neuronal nitric oxide synthase
NO: nitric oxide
NSAID: non‐steroidal anti‐inflammatory drug
NSCs: neural stem cells
NVU: neurovascular unit
O4: oligodendrocyte marker 4
Olig2: oligodendrocyte transcription factor 2
OMgp: oligodendrocyte myelin glycoprotein
OPCs: oligodendrocyte progenitor cells
Opn: osteopontin
OXPHOS: oxidative phosphorylation
PCs: pericytes
PD: Parkinson's disease
PDGF: platelet‐derived growth factor
PET: positron emission tomography
PI3K: phosphoinositide 3‐kinase
PLP: proteolipid protein
PNNs: perineuronal nets
PS1: presenilin 1
PTEN: phosphatase and tensin homolog
*PTSD*
: post‐traumatic stress disorder
RGCs: retinal ganglion cells
RhoA: ras homolog gene family member A
RNA: ribonucleic acid
SDF‐1α: stromal‐derived factor 1 alpha
SGZ: subgranular zone
SOCS3: suppressor of cytokine signaling 3
STAT: signal transducer and activator of transcription
STING: stimulator of interferon genes
SVZ: subventricular zone
TGF‐β1: transforming growth factor beta 1
TMEM164: transmembrane protein 164
TNFr‐1: tumor necrosis factor receptor 1
TNFα: tumor necrosis factor α
TSPO: translocator protein
VEGF: vascular endothelial growth factor
VEGF‐A: vascular endothelial growth factor A

## 1 INTRODUCTION

The intricate relationship between glial cells and neurons within the nervous system has long captivated neuroscientists. Over the years, research has illuminated the multifaceted nature of this relationship, from its pivotal role in the earliest stages of nervous system development to its profound impact on the progression of neurodegenerative and neuropsychiatric diseases. This review aims to provide a comprehensive exploration of glia–neuron interactions, traversing through the developmental milestones, maintaining homeostasis, contributing to neuronal vulnerabilities, and culminating in the damage of the nervous system in the forms of neurodegenerative and neuropsychiatric disorders.

The development of the nervous system is a testament to the intricate collaboration between glial cells and neurons. Indeed, glia orchestrate complex cellular interactions that sculpt neural circuits, laying the foundation for functional connectivity. Beyond development, glial cells assist in maintaining homeostasis and ensuring optimal neuronal function. Through mechanisms spanning neurotransmitter regulation, metabolic support, and synaptic pruning, glia is critical to the overall function of the nervous system (Allen & Lyons, 2018). Despite their resilience, neurons are not impervious to harm and several features predispose them to injury. Glial dysfunction or dysregulation can precipitate neuronal vulnerability, exacerbating the onset and progression of various neurological disorders. We delve into the intricate mechanisms underlying this delicate balance and the repercussions of its disruption. With respect to neurodegenerative and neuropsychiatric diseases, glial cells emerge as central players in the etiology and pathogenic cascade of such conditions.

Recognizing the pivotal role of glia in neurodegenerative and neuropsychiatric disorders, therapeutic strategies aimed at modulating glial function have garnered considerable attention. Given this, we discuss the evolving landscape of glia‐targeted interventions for mitigating neuronal degeneration and restoring neurological function. Overall, we believe a proper understanding of “building” the nervous system may provide the foundational knowledge required to design potential therapeutics to “rebuild” the nervous system.

## 2 GLIA CELLS CONTRIBUTE TO HEALTHY BRAIN DEVELOPMENT

**FIGURE 1:**
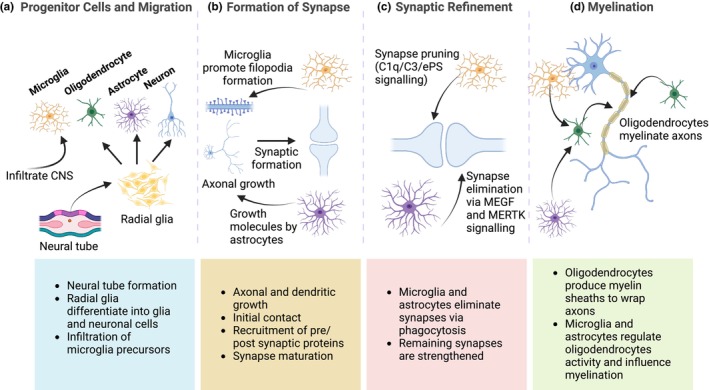
Key steps in CNS development and glial contributions. (a) Progenitor cells and migration. Radial glial cells originating from neural tube neuroepithelial cells generate various cell types, including neurons, astrocytes, and oligodendrocytes, which then migrate to their specified locations. Microglia precursors infiltrate the CNS. (b) Formation of synapses. Following dendritic and axonal growth, synaptic connections are formed. This process is promoted by microglia and astrocytes. (c) Synapse refinement. Synapses will then undergo either strengthening or elimination. Microglia and astrocytes contribute to synapse elimination through synapse engulfment. (d) Myelination. Oligodendrocytes provide myelin sheaths to wrap axons for proper conductance of electric signals in the nervous system. Microglia and astrocytes regulate this process as well. ePS: Exposed phosphatidylserine. Created with Biorender.com.

The development of the central nervous system (CNS) takes place in a highly orchestrated and tightly regulated manner which comprises several critical overlapping steps (Shonkoff & Phillips, 2000; Figure 1). As illustrated in Figure 1, several key steps are: (1) Neurulation that concludes with the formation of the neural tube and is characterized by a huge proliferative event, giving way to several progenitor cells. Progenitor cells will then differentiate into various cell types and migrate to their specific locations. (2) Neuronal differentiation and synapse formation following dendritic and axon growth. (3) Synapse refinement—Immature synapses undergo either elimination (in a process called “pruning”) or strengthening. (4) Myelination will allow for proper conductance of electric signals in the nervous system. Here we describe the crucial role of glial cells in the various steps of neurodevelopment.

### 2.1 Progenitor cells and migration

At the cellular level, the neural tube is formed of neuroepithelial cells which divide symmetrically to increase the number of cells (Figure 1a). Subsequently, these cells undergo a transformation into radial glial cells and thereby initiate the process of neurogenesis and gliogenesis that give rise to the progenitors of all neurons, oligodendrocytes, and astrocytes. Radial glial cells will undergo asymmetric division which will lead to differentiation of various cell types (reviewed by (Paridaen & Huttner, 2014)). The regulation of this process is defined by intrinsic and extrinsic signals which determine cellular fate. Along with neurogenesis and gliogenesis, migration of these cells would lead to the establishment of different laminae of the cortex (reviewed by (Buchsbaum & Cappello, 2019)). In contrast, microglia are derived from progenitors contained in the yolk sac and infiltrate the CNS around embryonic day 9 in rodents (Ginhoux et al., 2010; Gomez et al., 2014; Schulz et al., 2012).

### 2.2 Formation of synapses

Synapse formation may involve four successive stages: axonal and dendritic growth to appropriate target regions, generation of initial contact between axons and dendrites, development of pre‐ and postsynaptic specialization, and finally, synapse maturation (reviewed by (McAllister, 2007; Figure 1b)). The beginning of synapse formation is an initial contact between an axon and its postsynaptic counterpart. At first, the contacts formed between axonal and dendritic filopodia are unstable and transient; later some of them stabilize to give rise to a nascent synapse (Niell et al., 2004; Okabe et al., 2001; Sabo et al., 2006). The CNS has the capacity to respond to stimuli by modifying its shape and structure, a mechanism called structural plasticity. This feature allows drastic processes in CNS morphogenesis to take place including changes in dendritic morphology and turnover of axonal boutons (reviewed by (Forrest et al., 2018)). These events occur during synapse development in response to environmental factors and neural activity where synapses are being formed, stabilized, and eliminated (also see Section 2.3), but they can also take place across the lifespan triggered by learning, memory, and injury (see Sections 3 and 4; Schmidt et al., 2021; Holtmaat & Svoboda, 2009; Leuner & Gould, 2010; Tønnesen & Nägerl, 2016).

Microglia are proposed to play a critical role across both the formation and refinement (see Section 2.3) of synapses (reviewed in (Hammond et al., 2018)). Specifically, microglia promote spine formation in the developing somatosensory cortex of mice, particularly during the synaptogenic period between postnatal days 8 and 10 (Miyamoto et al., 2016). Using two‐photon imaging, it was observed that microglia establish contacts with dendrites and promote the formation of transient filopodia, which once stabilized could become a new synapse; depletion of microglia during this process resulted in the reduction of functional excitatory synapses, suggesting a critical role for these cells in synapse formation (Miyamoto et al., 2016). This is further supported by electron microscopy data highlighting the contact of microglia with spine head filopodia in mature synapses in the hippocampus (Weinhard et al., 2018). Given this, it is proposed that microglia help promote synapse formation during development. Furthermore, microglia promote excitatory synapse formation induced by learning in adults (Parkhurst et al., 2013), suggesting that microglia are involved in synapse formation both during development and adulthood. The role of microglia in synaptogenesis is not limited to excitatory synapses but extends to inhibitory synapses. Indeed, microglia promote growth and synapse formation in a particular axo‐axonic connection of chandelier cells (a type of GABAergic cortical interneuron) within the axon initial segment of neocortical pyramidal neurons during early postnatal development (Gallo et al., 2022).

In addition to microglia, astrocytes express cell‐adhesion molecules and secrete extracellular matrix proteins, growth factors, cytokines, and small extracellular vesicles that play a critical role in synaptic formation and axon growth (reviewed by (Hillen et al., 2018)). In rodents, synapse formation coincides with the differentiation and maturation of astrocytes consistent with their involvement in development (Chung et al., 2015). Indeed, the synaptogenesis period occurs in parallel with the development of astrocytes, with these cells contributing to synaptic maturation (Ullian et al., 2001). Astrocytes regulate different stages of synaptic formation along lifespan through a wide range of molecules, such as thrombospondins, glypicans, chordin‐like 1, cholesterol, and cell adhesion molecules (Allen et al., 2012; Baldwin & Eroglu, 2017; Blanco‐Suarez et al., 2018; Eroglu et al., 2009; Kucukdereli et al., 2011; Stogsdill et al., 2017; Ullian et al., 2001). In detail, immature astrocytes express high levels of thrombospondin during the first week of postnatal development, an extracellular matrix protein that plays a critical role in synapse formation in the cerebral cortex (Christopherson et al., 2005). Once the expression of this protein declines, astrocytes begin to secrete proteins that positively (e.g., Hevin) or negatively (e.g., SPARC) modulate synaptogenesis (Chung et al., 2015; Farhy‐Tselnicker & Allen, 2018). In addition, astrocytes promote AMPA receptor localization at the synapse through the secretion of heparan sulfate proteoglycans 4 and 6 (Gpc4/6; Allen et al., 2012). Astrocytes secrete transforming growth factor beta 1 (TGF‐β1) which regulates the formation of excitatory and inhibitory synapses and, in turn, the excitatory and inhibitory balance (Diniz et al., 2012, 2014). In line with these findings, it has been shown that astrocytes secrete small extracellular vesicles that contain fibulin‐2, which regulates spine formation through TGF‐β1 (Patel & Weaver, 2021).

### 2.3 Synaptic refinement

Following synapse formation both microglia and astrocytes play a role in synapse refinement via pruning events (Figure 1c). Microglia are critical for regulating the number of synapses as they play a role in synaptic pruning. For instance, mice with a transient reduction of microglia in the hippocampus during postnatal development showed delayed synaptic pruning which resulted in an excess of immature synaptic connections (Paolicelli et al., 2011). Microglial pruning of synapses is linked to synaptic activity (Tremblay et al., 2010) and is mediated by signals including components of the complement cascade such as C1q and CR3/C3 (Schafer et al., 2012; Stevens et al., 2007) and phosphatidylserine (Li et al., 2020a; Scott‐Hewitt et al., 2020). It has been described that astrocytes also play an important role in the elimination of synapses during development and in synaptic plasticity. These cells have been proposed to present a phagocytic capacity that relies on mechanisms different from those of microglia (activation of MEGF10 and MERTK pathways) to mediate synapse elimination (Chung et al., 2013).

### 2.4 Myelination of the nervous system

As synapses are being formed and refined, oligodendrocytes are responsible for the myelination process, both in white and gray matter (Figure 1d). Upon reaching maturation, oligodendrocytes produce myelin sheaths which wrap the axons and facilitate rapid nerve conduction. This process promotes the propagation of action potentials by reducing membrane capacitance (Huxley & Stämpeli, 1949; Rushton, 1951). In the developing nervous system, certain regions of the peripheral nervous system (PNS) myelinate first, followed by the spinal cord and, finally, the brain. The control of myelination during development is regulated by many intrinsic and extrinsic signals both spatially and temporally (reviewed by (Nave & Werner, 2014)). These signals include soluble factors, such as growth factors and extracellular matrix molecules, cell components including kinases, cell adhesion molecules, actin cytoskeleton, and transcriptional and translational regulation in oligodendrocytes. (Bauer et al., 2009; Bercury & Macklin, 2015; Dimas et al., 2019; Calver et al., 1998; Musah et al., 2020; Ornelas et al., 2020).

Interestingly, microglia and astrocytes may also play a role in myelination. With respect to microglia, the role of neuronal activity‐regulated microglia in modifying developmental myelination by oligodendrocytes has been demonstrated in a zebrafish model (Hughes & Appel, 2020). Furthermore, the absence of microglia can induce structural changes in myelin leading to poor cognitive flexibility in mice (McNamara et al., 2023). These results suggest that activity‐regulated microglia may play a role in influencing myelination to strengthen cognitive circuits, given the strong link between neuronal activity and myelination. As for the contribution of astrocytes to this process (reviewed by (Stogsdill et al., 2023)), platelet‐derived growth factor (PDGF) and C‐X‐C Motif Chemokine Ligand 1 (CXCL1) produced by white matter astrocytes may work together to regulate when and where oligodendrocyte progenitor cells (OPCs) migrate and proliferate (Tsai et al., 2002). In line with these findings, semaphorins derived from astrocytes promote the displacement of OPC from their close proximity to blood vessels to allow their differentiation during development (Su et al., 2023). Additionally, it has been demonstrated that the cytokine leukemia inhibitory factor (LIF) released by cultured astrocytes in the presence of neuronal activity improves myelination (Ishibashi et al., 2006). Astrocytes also provide oligodendrocytes with lipids allowing myelination during development (Camargo et al., 2017). The coordination of these astrocyte‐derived signals with activity‐dependent neuronal signals ultimately dictates the timing and extent of developmental myelination, as well as the fine‐tuned process of adaptive myelination during learning.

## 3 THE ROLE OF GLIAL CELLS IN ADULT BRAIN HOMEOSTASIS, PLASTICITY, AND NEUROGENESIS

### 3.1 Homeostatic and metabolic processes

**FIGURE 2:**
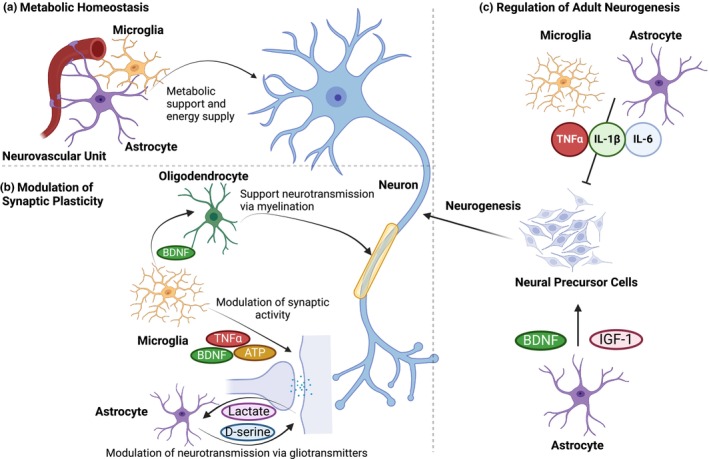
The role of glial cells in adult brain homeostasis, plasticity, and neurogenesis. (a) Glial cells regulate the blood–brain barrier and neurovascular unit and are functionally coupled to neurons to sustain metabolic homeostasis. (b) Glial cells contact neurons to support synaptic function and plasticity. Synaptic functions can be controlled by microglia via the release of cytokines and trophic factors, and astrocytes through the regulation of gliotransmitters such as D‐serine and lactate. Oligodendrocytes further contribute to proper neuronal function as they can modulate axonal conduction by controlling myelin thickness and regulating Ranvier nodes. (c) The potential for adult neurogenesis is tightly regulated by glial cells in an inhibitory manner by pro‐inflammatory cytokine release or in a supportive manner by the release of neurotrophic factors. Created with Biorender.com.

Among all organs in the human body, the brain is one of the highest consumers of energy. The continuous neuronal activity together with high‐demanding functions, such as synaptic transmission and production of neurotransmitters, leaves the brain dependent on an uninterrupted supply of energy (reviewed by (Attwell & Laughlin, 2001). This influx is mainly regulated through dynamic interactions between neurons, glial cells and vasculature (reviewed by (Bonvento & Bolaños, 2021; Hösli et al., 2022)) (Figure 2a). While glycolysis provides a source of energy, mature neurons rely mainly on oxidative phosphorylation (OXPHOS) to meet their high energetic demands (Hertz et al., 2007; Sokoloff et al., 1977; Whitesell et al., 1995; Zheng et al., 2016). This energy supply supports excitability, biosynthesis of neurotransmitters, synaptic transmission and plasticity (reviewed by (Dienel, 2012)). When energy supply fails, for example through peripheral metabolic challenges, vasculature failure, or astrocytic pathology, brain metabolism and subsequent neurotransmission are at risk. Therefore, complementary energy sources can be used under acute phases of peripheral metabolic stress (e.g., glucose deprivation), including ketone bodies (Courchesne‐Loyer et al., 2017; Jensen et al., 2020) and pyruvate (Gonzalez et al., 2005).

Connected to the vasculature, astrocytes sustain glycolytic function, maintaining their own needs and regulating energy toward neurons and synapses (Hösli et al., 2022). Astrocytes are the main cell type responsible for fulfilling the metabolic needs of the brain. They act as the main reservoirs of glycogen (Calì et al., 2019), which in turn could be used to produce lactate as a form of energy during higher energy demands (Barros & Weber, 2018; Pellerin & Magistretti, 2004; Schurr et al., 1998). During the last decade, the importance of the astrocyte‐neuron lactate shuttle has been debated, questioning whether lactate production in astrocytes could support high‐demand neuronal processes and/or support specific groups of neurons (Chi & Roberts, 2003; Chih et al., 2001; Mangia et al., 2009; Mason, 2017). While astrocytes remain the primary glycolytic cells of the brain (Supplie et al., 2017), recent studies have shown the ability of astrocytes to metabolize fatty acids in order to sustain cognitive function (Morant‐Ferrando et al., 2023).

### 3.2 Blood–brain barrier and neuron–glia vascular coupling

The blood–brain barrier (BBB) is a specialized vasculature that controls brain homeostasis and transport of metabolites and restricts access of peripheral cells to the brain parenchyma (reviewed by (Kugler et al., 2021); Figure 2a). Strict control of the bi‐directional movement of molecules across the BBB is provided by specialized brain endothelial cells (ECs) that form tight junctions structurally dependent on the expression of certain proteins, for example, claudins, occludin, and zonula occludens, enriched in the CNS compared with non‐neural tissues, suggestive of their importance for BBB integrity (reviewed by (Daneman & Prat, 2015; Luissint et al., 2012)). More recently, the role of the neurovascular unit (NVU) in the regulation of brain metabolism has been established. The NVU is an ensemble of endothelial cells, neurons, pericytes, glia, smooth muscle cells and extracellular matrix (reviewed by (Lok et al., 2007)). Close interaction of these cell types allows for intensification of cerebral blood flow (CBF) following mental or physical activity in a process called neurovascular coupling (reviewed by (McConnell et al., 2017)). Neurons can directly initiate the local vascular response via the release of neurotransmitters such as glutamate leading to the activation of enzymes such as neuronal nitric oxide (NO) synthase (nNOS) and cyclooxygenase 2 (COX‐2) in postsynaptic neurons, which produce potent vasodilators (NO and prostanoid, respectively) (Attwell et al., 2010; Lecrux & Hamel, 2016). At the same time, glutamate acts on metabotropic glutamate receptors in glial cells, triggering the production of vasoactive agents (reviewed by (Benarroch, 2005)).

Glia within the NVU are crucial components that ensheathe the endothelium forming a so‐called secondary barrier, and nutrients required for CNS function are delivered from the blood vessels to neurons mainly via glia cells (reviewed by (Hurley et al., 2015)), while waste compounds are passed via glial cells to microglia or back into the bloodstream (reviewed by (Marina et al., 2018)). Astrocytes not only regulate neuronal homeostasis and contribute to plasticity (de Pittà et al., 2016; Sofroniew & Vinters, 2010), but also contact blood vessels inducing either vasoconstriction (e.g., by arachnoid acid) or vasodilation (e.g., by prostaglandins; reviewed by (Kimelberg, 2010)). Astrocytes physically interact with BBB and functionally contribute to its permeability for the traffic of molecules such as glucose and proteins (reviewed by (Abbott et al., 2006)). Similar functions are performed in the retina by Müller glia, which contact both neurons and blood vessels (reviewed by (Subirada et al., 2018)). Glial cells also play an active role in facilitating vascular angiogenesis via the expression of factors, such as VEGF and TGF‐β1 in radial glia cells (Siqueira et al., 2018). Radial glia cells are pivotal for the formation of the NVU, as they both give rise to neurons and astrocytes during development (da Silva et al., 2019) and support BBB maturation through the secretion of factors such as retinoic acid, which increases the expression of BBB‐specific genes in ECs (Mizee et al., 2013). This is a two‐way interaction, as ECs can increase glial fibrillary acidic protein (GFAP) expression in radial glia in a VEGF‐A‐dependent manner, leading to astrocyte differentiation and NVU formation (da Silva et al., 2019).

Another crucial component of the BBB is pericytes (PCs), which are in direct contact with ECs and surrounded by basal lamina. A plethora of studies show the role of PCs in the regulation of blood flow, transport of substances between the brain and bloodstream (reviewed by (Kugler et al., 2021)), and the establishment and maintenance of the BBB (reviewed by (Brown et al., 2019)). Within the brain, PCs can actively relax or contract to change CBF in response to localized changes in neuronal activity (Hall et al., 2014; Rucker et al., 2000). PCs play a critical role in BBB establishment as they are required for the formation of functional EC tight junctions. This occurs possibly by a mechanism that suppresses genes known to be involved in vascular permeability such as angiopoietin‐2, plasmalemma vesicle‐associated protein, and leukocyte adhesion molecules (LAMs) (Daneman et al., 2010). The dynamic nature of the BBB, which can be modulated by multiple signals derived from various cell types, is crucial to enabling the communication between the CNS and the periphery (reviewed by (Segarra et al., 2021)).

### 3.3 Synaptic plasticity

Synaptic plasticity refers to the ability of neurons to make long‐lasting changes that take place in the synapses of a particular network. It involves structural and functional changes that modify the strength or efficacy of synaptic transmission (reviewed by (Citri & Malenka, 2008)). In mammals, different forms of synaptic plasticity have been described, with short‐term and long‐term plasticity as the prominent ones. Short‐term plasticity, ranging from milliseconds to several minutes, is mainly associated with changes in presynaptic neurotransmitter release, and seems to be relevant for transient changes in behavior and sensory input adaptations (reviewed by (Blitz et al., 2004)). Long‐term synaptic plasticity is associated with long‐lasting modifications in synaptic strength through pre‐ and/or postsynaptic mechanisms, which may accompany structural alterations such as changes in dendritic spine density and actin polymerization (reviewed by (Herring & Nicoll, 2016; Holtmaat & Svoboda, 2009)). These activity‐dependent changes are bidirectional, allowing long‐term potentiation (LTP) or depression (LTD) of stimulated connections. LTP mechanisms have been extensively studied as they are critical to the cellular and molecular machinery underlying memory formation (Martin et al., 2000; Pastalkova et al., 2006; Whitlock et al., 2006). In addition to LTP and LTD, it is important to highlight the idea of homeostatic plasticity. Homeostatic plasticity refers to a set of mechanisms that maintain the stability of neuronal functions through coordinated plasticity among cellular and subcellular compartments, acting to stabilize the activity of a neuron or neuronal circuit around some set‐point value (Turrigiano & Nelson, 2004). Indeed, a form of homeostatic plasticity seems to be relevant in mature circuits as prolonged changes in activity result in synaptic adaptations (reviewed by (Turrigiano, 2012)).

For several years, experience‐dependent alterations in the strength of synapses were focused on neuronal activity. However, the majority of synapses in the CNS are contacted by astrocytes and other cell types, having a morphofunctional organization that characterizes multipartite synapses (reviewed by (Araque et al., 1999; Farhy‐Tselnicker & Allen, 2018; Semyanov & Verkhratsky, 2021)) (Figure 2b). Astrocytes play a prominent role in regulating synaptic activity through the release of gliotransmitters, such as D‐serine and lactate, and this process is bidirectionally regulated between neuronal activity and astrocytic activation (Abreu et al., 2023). Moreover, astrocytes participate in the clearance of neurotransmitters, regulating neuronal excitability in both short‐term and long‐term synaptic plasticity (Goubard et al., 2011; Murphy‐Royal et al., 2017; Perez‐Alvarez et al., 2014; Sibille et al., 2014). Importantly, and as referred previously in Section 2.3, astrocytes phagocytose adult hippocampal synapses, which maintains proper hippocampal synaptic connectivity and plasticity (Lee et al., 2021). Lastly, it is worth noting that neuronal activity can affect the transcriptome of astrocytes (Hrvatin et al., 2018) and such transcriptional changes are sufficient to modulate circuit activity (Huang et al., 2020).

Microglia have also been shown to play an important role in synaptic plasticity (reviewed by (Andoh & Koyama, 2021)). By releasing several cytokines and neurotrophic factors such as tumor necrosis factor α (TNFα), ATP, or brain‐derived neurotrophic factor (BDNF), microglia contribute to neuronal excitability, partaking in the reorganization of synaptic receptors in neurons and glutamate release by astrocytes (Parkhurst et al., 2013; Pascual et al., 2012; Santello et al., 2011; Stellwagen et al., 2005). In addition, microglia regulate synaptic plasticity by removing synapses via phagocytosis, a mechanism that seems to be associated with synaptic weakening and LTD (Schafer et al., 2012; Zhang et al., 2014).

Oligodendrocytes also participate in synaptic plasticity through the regulation of axonal transmission efficiency. This is achieved by two mechanisms: (1) controlling myelin thickness and (2) regulating the structure of Ranvier nodes (Pajevic et al., 2014; Sinclair et al., 2017). As oligodendrogenesis persists into adulthood, OPCs receive excitatory and inhibitory signals and modulate circuit activity accordingly (Bergles et al., 2000; Lin & Bergles, 2004). Moreover, activity is required to maintain myelination (Sinclair et al., 2017) and microglia could also regulate this process via BDNF release (Geraghty et al., 2019). These data suggest that activity‐dependent regulation of myelin contributes to adaptive neuronal function. Taken together, microglia, astrocytes, and oligodendrocytes work together to contribute to synaptic plasticity.

### 3.4 Regulation of adult neurogenesis

Adult neurogenesis is a well‐known phenomenon that involves the generation of new neurons through the differentiation of neural stem cells (NSCs) during adulthood (Figure 2c). In mammals, adult neurogenesis is regulated by a complex interplay of molecular and environmental factors, and this process is generally restricted to two canonical sites: the subgranular zone (SGZ) of the dentate gyrus and the subventricular zone (SVZ) of the lateral ventricles (Ming & Song, 2011). However, other putative neurogenic sites have been suggested such as the striatum and the hypothalamus (Bartkowska et al., 2023; Ernst et al., 2014). Although low levels of hippocampal neurogenesis are still retained in healthy, aged individuals (Moreno‐Jiménez et al., 2019), recent work has demonstrated an age‐associated decline in adult neurogenesis in parallel with decreased cognitive abilities in humans (Babcock et al., 2021; Boldrini et al., 2018; Klempin & Kempermann, 2007). Given this, it is important to consider the mechanisms that regulate adult neurogenesis and their potential for therapeutic implications. The lineage specification, proliferation, and differentiation of NSCs in the adult brain are regulated by both intrinsic factors such as gene expression, growth factors, and signaling molecules and extrinsic factors such as environment, physical activity, and pharmacological agents (Ming & Song, 2011).

Both microglia and astrocytes have been shown to secrete various factors that can affect neurogenesis (Quesseveur et al., 2013; Sato, 2015; Sultan et al., 2015). Key secretory molecules released by microglia such as BDNF (Nakajima et al., 2001) and insulin‐like growth factor‐1 (IGF‐1) (Suh et al., 2013) are capable of impacting neurogenesis. Accordingly, a hippocampal injection of BDNF is sufficient to increase the number of newborn neurons (Scharfman et al., 2005). Furthermore, IGF‐1 has been shown to positively regulate adult neurogenesis (Åberg et al., 2003; Yuan et al., 2015). On the other hand, pro‐inflammatory cytokines released from microglia have an inhibitory effect on neurogenesis (Battista et al., 2006; Borsini et al., 2015). Consistent with this, deletion of proinflammatory TNF‐α or TNFr‐1 in animals is associated with increases in hippocampal neurogenesis (Chen & Palmer, 2013).

In addition to microglia, astrocytes may also promote neurogenesis through the release of BDNF, fibroblast growth factor‐2 (FGF‐2), ciliary neurotrophic factor (CNTF), and D‐serine. BDNF overexpression in mouse astrocytes was shown to be sufficient to drive neurogenesis (Quesseveur et al., 2013). Similarly, enhancement of hippocampal neurogenesis has also been attributed to FGF‐2 release from astrocytes (Kirby et al., 2013). Astrocytes have also been shown to regulate adult neurogenesis, NSC proliferation, and differentiation through the expression of CNTF that interacts with its receptor CNTFRα, predominantly expressed in neural progenitor cells (Ding et al., 2013; Pasquin et al., 2015). In addition to trophic factor release, astrocytes may also play a role in neurogenesis via the release of metabolites such as D‐serine. Indeed, blocking vesicular release of such metabolites from astrocytes results in decreased levels of maturation, survival, and synaptic integration of newborn neurons (Sultan et al., 2015). Considering our current understanding of neurogenesis, strategies to increase new neurons remain extremely limited which leaves this cell population vulnerable to injury. Targeting glia may represent a valuable approach to enhancing adult neurogenesis.

## 4 SUSCEPTIBILITY FOR NEURONAL INJURY AND LOW RECOVERY POTENTIAL MEDIATED BY GLIA

### 4.1 Susceptibility for neuronal injury

**FIGURE 3:**
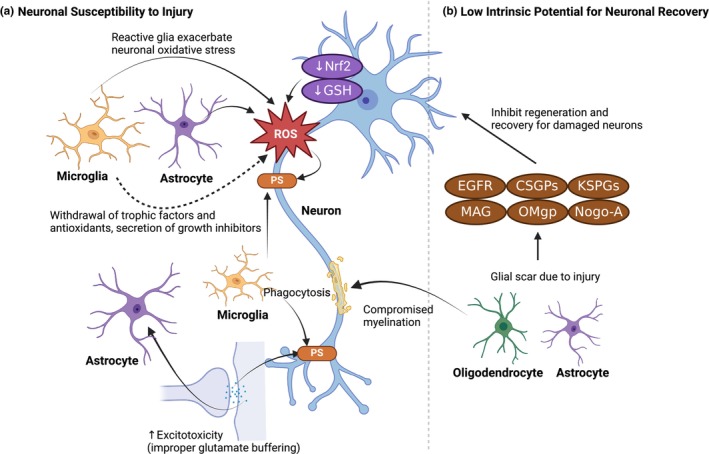
Susceptibility for neuronal injury and low recovery potential mediated by glia. (a) Reactive microglia and astrocytes contribute to neuronal susceptibility to neuronal injury by withdrawing supportive factors such as trophic factors and antioxidants and secreting growth inhibitors, which further exacerbate neuronal sensitivity to oxidative stress. Improper glutamate buffering by astrocytes may also amplify neuronal susceptibility to excitotoxic insult. Additionally, microglial phagocytosis may increase because of increased phosphatidyl serine exposure. (b) During injury or disease, secreted factors from glial scars result in the upregulation of several inhibitory molecules, which further compromise the ability for regeneration and recovery of damaged neurons. Oligodendrocytes also increase neuronal susceptibility as they may withdraw key myelinating activities during injury or disease. GSH, glutathione; PS, phosphatidylserine; ROS, reactive oxygen species. Created with Biorender.com.

Neurons have unique properties which increase their susceptibility to damage and injury (Figure 3a). In the adult CNS, neurons must carry out neurotransmission over their highly complex arborization which requires propagation of electrical activity across long distances. This may result in neurons being predisposed to potential excitotoxic insults as well as oxidative injury given high metabolic demands to support the energy‐consuming neurotransmission activity. Post‐mitotic neurons of the CNS show little regenerative capacity as adult neurogenesis is a limited process in the CNS. Given this, neurons accumulate stress throughout a lifetime and may become compromised as the brain ages. Indeed, excitotoxicity, a harmful process in neurons caused by excessive glutamate signaling, leads to neuronal death after injury (Guerriero et al., 2015; Gwag et al., 1995).

Hyperexcitability is present in several diseases of the nervous system including Alzheimer's disease (AD) (Šišková et al., 2014), Parkinson's disease (PD) (Kamble et al., 2022), amyotrophic lateral sclerosis (ALS) (Do‐Ha et al., 2018), and Huntington's disease (HD) (Cummings et al., 2009). Increased extracellular glutamate results in aberrant sodium and calcium entry into the cell, a process which is mainly controlled by N‐methyl‐d‐aspartate (NMDA) receptors (Tymianski et al., 1993). Neurons are well equipped with mitochondria at synaptic sites to deal with this calcium influx. However, excessive mitochondrial calcium handling can perturb respiration, resulting in the production of reactive oxygen species (Reynolds & Hastings, 1995). In addition, increased mitochondrial calcium can also elicit pore opening which results in neuronal death (Stout et al., 1998). Interestingly, glial cells may exacerbate excitotoxic events in the vulnerable neuronal population.

During normal physiological conditions, microglia and astrocytes support neurons by secreting growth factors, providing metabolic support, and promoting synaptic function and plasticity (reviewed by (Reemst et al., 2016; Verkhratsky & Nedergaard, 2018)). During injury, neurodegenerative, or neuropsychiatric conditions, supportive functions are withdrawn and glial cells can take on a reactive, non‐physiological phenotype. In several neurodegenerative diseases, astrocytes lose their capability to properly buffer glutamate which may further lead to excitotoxic events in neurons (Behrens et al., 2002; Howland et al., 2002; Li et al., 2019). Interestingly, neurons stressed by glutamate may expose phosphatidylserine because of the inhibition of phosphatidylserine translocases by oxidative stress, which could lead to aberrant phagocytosis of neurons by microglia (Neher et al., 2013; Suzuki et al., 2013). This suggests that oxidative stress results in a phagocytic signal on the neuronal surface, and subsequent microglial phagocytosis could contribute to neuronal loss and synaptic dysfunction. Therefore, glia during injury or in disorders of the nervous system may contribute to excitotoxic outcomes experienced by already vulnerable neurons.

Another characteristic of neurons that predisposes them to injury is their high metabolic needs, in which OXPHOS is the main energy source. Indeed, the majority of the ATP generated by neurons in the CNS is to maintain functional synapses and promote neuronal excitability (Rangaraju et al., 2014). Interestingly, undifferentiated NSCs rely on glycolysis, and during the differentiation process, metabolism switches towards OXPHOS (Mandal et al., 2011; Prigione et al., 2010). Once post‐mitotic, neurons are prone to oxidative stress given their high mitochondrial activity needed to sustain neurotransmission. Further predisposing neurons to oxidative stress is their relatively low capacity for redox homeostasis as compared to astrocytes (Bolaños et al., 1995; Dringen et al., 1999). Neurons have low levels of the transcription factor Nrf2, which is required to produce glutathione. Therefore, they rely mainly on astrocytic‐derived glutathione (Jimenez‐Blasco et al., 2015; Raps et al., 1989). Moreover, under stress conditions, both microglia and astrocytes have been shown to release oxidative molecules which may promote axonal damage and exacerbate neuronal loss (Bido et al., 2021; Chun et al., 2020; Michaels et al., 2020; Muñoz et al., 2018). Astrocytes may further exacerbate oxidative insults during disease or injury as they can withdraw their supportive antioxidant function. Together, these results indicate that neurons are intrinsically sensitive to oxidative damage and that glia may further exacerbate this oxidative stress, ultimately resulting in neuronal loss.

### 4.2 Low intrinsic potential of neurons for recovery

The ability of the CNS to effectively transmit signals is frequently compromised when nerve cells experience damage, whether from direct physical trauma or from disease‐associated events. While in higher organisms the PNS can undergo successful repair, the CNS shows comparatively restricted recovery (reviewed by (Steward et al., 2013)). Additionally, as described above, mammals exhibit a limited spatial distribution of neurogenic areas. The regenerative potential of the adult mammalian CNS is restricted by both intrinsic neuronal factors and extrinsic inhibitory factors originating from glial cells (reviewed in (Fawcett, 2020)) (Figure 3b). After a CNS injury, a variety of cells, including microglia and astrocytes, converge at the injury site to create a glial scar. While the formation of this scar is vital to address immediate damage, it may also establish a barrier that impedes axon regrowth (reviewed in (Cooke et al., 2022)). Inhibitory molecules associated with myelin and the glial scar such as myelin‐associated glycoprotein (MAG), Nogo‐A, and oligodendrocyte myelin glycoprotein (OMgp), account for the inhibitory activity in the CNS. For instance, the activity of integrins, which can promote neurite growth in both embryonic and adult neurons (Cheah et al., 2016; Gardiner et al., 2005; Neugebauer et al., 1991), is suppressed by the presence of Nogo‐A (Hu & Strittmatter, 2008). From a mechanistic standpoint, epidermal growth factor receptor (EGFR) has been demonstrated to be a modulator of regeneration as it can respond to several molecules secreted by glia during injury. Consistent with this, activation of EGFR has been reported during inhibition of regeneration, and blocking EGFR activity is sufficient to drive regeneration of damaged nerve fibers (Koprivica et al., 2005). Modulating EGFR signaling in neurons may help alleviate regenerative suppression caused by glial secretory molecules.

Another factor contributing to low regenerative ability is the development of extracellular matrix structures called perineuronal nets (PNNs), which are composed of chondroitin sulfate proteoglycans (CSPGs) (reviewed by (Fawcett et al., 2022)). Astrocytes play a critical role in the formation of PNNs as they have been shown to secrete many of the key cellular components involved in this highly organized extracellular matrix structure (reviewed by (Dzyubenko et al., 2016)). These nets are mesh‐like matrix layers that surround the soma and dendrites. They participate in the stabilization of synapses and connections and play an important role in neural plasticity (Hockfield et al., 1990; Pizzorusso et al., 2006). However, CSPGs have been shown to constrain axonal growth during post‐injury periods (Imagama et al., 2011; Jones et al., 2003). Conversely, the use of chondroitinase to break down these inhibitory CSPGs has been shown to enhance axonal sprouting and functional recovery in rodent models of spinal cord injury (Rosenzweig et al., 2019). Moreover, different types of proteoglycans can distinctively modulate axonal growth and may contribute to the impairment in axon regeneration. Indeed, cut dopaminergic nigrostriatal axons exhibit extensive sprouting in regions containing heparan sulfate proteoglycans (HSPGs), but fail to extend into adjacent areas containing CSPGs and keratan sulfate proteoglycans (KSPGs), which suggests that the regrowth of severed CNS axons may be locally facilitated by HSPGs but hindered by CSPGs and KSPGs (Moon et al., 2002).

In a mouse model using glial scar‐free microlesions, it was found that most cortical axons are unable to initiate a regenerative response after injury, even in the absence of a lasting glial reaction at the lesion site. This indicates that, in this model, the primary constraint to neuronal recovery is not because of glial‐dependent inhibition, but rather an inability of neurons to resume axonal growth (Canty et al., 2013). The ability for axons to grow in the CNS is markedly higher during the embryonic stage than in adulthood, suggesting that, as they mature, CNS axons diminish their regenerative potential. Notably, embryonic neurons survive, integrate, and show substantial growth and functional impact when implanted into the adult CNS, regardless of the inhibitory conditions (Gaillard et al., 2007; Lu et al., 2012). The contrasting capabilities between embryonic and mature neurons might be attributed to the downregulation of genes required for regeneration. Multiple signaling mechanisms involved in regulating neurite elongation are downregulated in adulthood, perhaps to mitigate abnormal growth (reviewed in (Varadarajan et al., 2022)); however, this downregulation can also preclude repair and regeneration in mature organisms.

Taken together, neurons display intrinsic properties that predispose them to damage while having relatively low regenerative capacity. Glial cells can exacerbate this damage and inhibit their regeneration. Given this, targeting glia and reinitiating some of the programs seen during development may represent a viable therapeutic strategy to reduce the susceptibility of neurons to injury and increase their regenerative capacity.

## 5 GLIAL CELLS CONTRIBUTE TO NEURODEGENERATION AND NERVOUS SYSTEM DYSFUNCTION

### 5.1 Neurodegenerative diseases: Alzheimer's disease and Parkinson's disease

**FIGURE 4:**
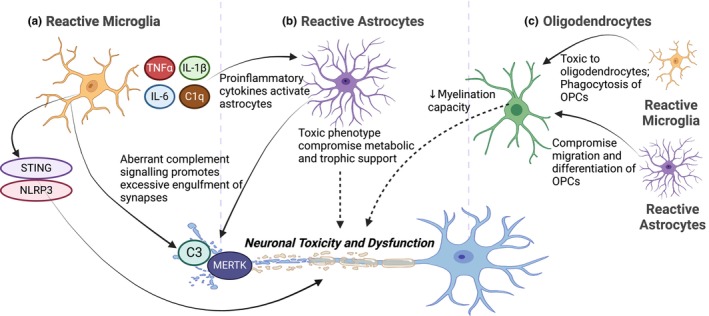
Glial cells contribute to neurodegeneration and nervous system dysfunction. (a) Reactive microglia present in neurodegenerative or neuropsychiatric diseases contribute to neuronal loss and dysfunction by secreting pro‐inflammatory cytokines and activating pro‐inflammatory signaling pathways such as STING and NLRP3. Microglia also contribute to synapse loss and dysfunction through aberrant complement‐mediated phagocytosis. (b) Astrocytes become reactive during neurodegeneration and in neuropsychiatric diseases and this toxic phenotype results in compromised metabolic/trophic support and increased pro‐inflammatory signaling and phagocytosis. (c) Oligodendrocytes affected by disorders of the nervous system reduce their ability to properly myelinate axons, a process that is further disrupted by reactive microglia or astrocytes. Created with Biorender.com.

As described above, there is a relatively low potential for adult neurogenesis, resulting in little turnover of neurons (Figure 3b). Given this, existing neurons are left susceptible to injury over long periods that span a lifetime and have very low ability to recover. Considering this, it is important to understand how glial cells may exacerbate neuronal stress in both disease and dysfunction to better understand how to protect these sensitive neurons (Figure 4). AD and PD are the two most common neurodegenerative disorders, with AD being typically characterized by cognitive decline and PD by symptoms that primarily affect the motor system. Although neuronal death and loss of synapses are the key molecular features that precipitate these symptoms, the contribution of glia to disease progression has recently gained attention. Aging is a major risk factor for both AD and PD, with disease prevalence increasing in older populations. Glial cells undergo transcriptional changes with aging, including widespread inflammatory gene alterations in microglia, and regional changes in astrocytes and oligodendrocytes, especially in the hippocampus and substantia nigra (Soreq et al., 2017). These age‐related glial changes may contribute to neuronal vulnerability seen in AD and PD. Traditionally, glial changes have been labeled as activated or reactive, but this oversimplifies the underlying complexity. Activated/reactive microglia and astrocytes exhibit heterogeneous molecular, morphological, and functional states that are not necessarily neurotoxic (reviewed by (Escartin et al., 2021; Paolicelli et al., 2022)). Here we refer to activated or reactive glia as cells in specific states that may contribute to disease pathology, and we propose a framework for therapeutic strategies based on these insights (see Section 6).

#### 5.1.1 Complement signaling

The complement cascade has been shown to be activated in a wide range of neurodegenerative diseases, including AD and PD (reviewed by (Brennan et al., 2016)). More specifically, examination of post‐mortem human brains has demonstrated that complement proteins are associated with amyloid‐rich plaques in AD (Eikelenboom & Stam, 1982) and α‐synuclein inclusions in PD (Loeffler et al., 2006; Yamada et al., 1992). Several groups have demonstrated that both Tau and amyloid beta are sufficient to activate the complement cascade in models of AD (Rogers et al., 1992; Shen et al., 2001; Sjöberg et al., 2008). Additionally, both neurotoxin and α‐synuclein PD animal models show increased complement activity (Jing et al., 2021; Rocha et al., 2015). It is worth noting that astrocytes may also be involved in complement signaling in neurodegenerative diseases. For example, animals injected with preformed α‐synuclein fibrils show increased expression of astrocytic C3, which may induce neuronal cell death, suggesting a role of astrocyte‐complement signaling in non‐cell autonomous neurodegeneration (Ma et al., 2021). In addition, in a TauP301S mouse model of AD, it was determined that astrocytes contribute to synapse elimination via C1q‐dependent activity (Dejanovic et al., 2022). Finally, activation of the complement system within microglia contributes to synapse loss in AD (Hong et al., 2016) and damaged dopaminergic neurons can activate the microglial complement receptor C3aR to produce increased complement C1q which may contribute to synaptic abnormalities and neurodegeneration in PD (Rocha et al., 2015; Zhang et al., 2023b).

#### 5.1.2 Activated microglia and pro‐inflammatory conditions

It has been suggested that dysfunctional microglia are involved in the development of neurodegenerative diseases. Microglial activation states have been strongly correlated with AD and found to be present in post‐mortem AD brains in which microglia are concentrated near plaques (Felsky et al., 2019; Itagaki et al., 1989; McGeer et al., 1987). Furthermore, it has been reported in AD brains that the level of microglia activation correlates with Tau and amyloid levels (Dani et al., 2018; Hayes et al., 2002). Transcriptomic analyses from both mouse models and human brains have revealed that reactive microglia phenotypes are associated with AD progression (Friedman et al., 2018; Mathys et al., 2017). Similarly, activated microglia have also been described in PD post‐mortem brains and mice expressing α‐synuclein (McGeer et al., 1988; Su et al., 2008).

In AD and PD, microglia release a wide number of pro‐inflammatory cytokines creating robust inflammatory states (reviewed by (Badanjak et al., 2021; Wang et al., 2015)). Consistent with this, AD patients display higher levels of released pro‐inflammatory cytokines compared to healthy controls (Swardfager et al., 2010). Furthermore, in AD brains, translocator protein (TSPO) PET signals are increased, which is a proxy for increased levels of neuroinflammation (Diorio et al., 1991; Venneti et al., 2006). In addition, amyloid β (Aβ) is sufficient to increase pro‐inflammatory mediators in in vitro models of AD (Combs et al., 2001; Del et al., 1995). In rodent models of AD, neuroinflammation is also observed because of an increase in several pro‐inflammatory cytokines (Hanzel et al., 2014; Janelsins et al., 2008; Martin et al., 2017). Such cytokines released by activated microglia are sufficient to produce neuronal toxicity (Liddelow et al., 2017). Taken together, microglia may adopt a pro‐inflammatory phenotype that may be a key contributor to neuronal loss in AD.

Similar to AD, several lines of evidence point to a role for microglia and neuroinflammation in PD. Post‐mortem idiopathic PD brains display both increased amounts of microglia and more activated microglia, as determined by an amoeboid morphology, when compared to age‐matched control brains (Smajic et al., 2022). An animal model of PD in which α‐synuclein was ectopically expressed showed transcriptomic changes in microglia as marked by CD11b+ reactivity in a region‐specific manner (Basurco et al., 2023). Specifically, bulk RNA sequencing from the PD mouse model brain revealed that microglia from the midbrain did not show proinflammatory signatures, whereas striatal microglia showed pro‐inflammatory features consistent with disease‐associated microglia (Basurco et al., 2023). Additionally, some findings demonstrate a significant age‐related correlation of T cell numbers with dopaminergic nerve terminal loss in α‐synuclein mice as compared to wild‐type controls (Rauschenberger et al., 2022). Considering this, it is plausible that microglia produce pro‐inflammatory effects on the dopaminergic synaptic terminals in the striatum rather than the dopaminergic cell bodies in the midbrain.

Regardless of the location of action, microglia seem to contribute to neuronal loss in PD. One line of evidence suggests that microglial exposure to α‐synuclein results in stimulator of interferon genes (STING)‐dependent interferon release, which may mediate neurodegeneration (Hinkle et al., 2022). The activity of microglial NLR Family Pyrin Domain Containing 3 (NLRP3), a cytosolic pattern recognition receptor that acts as the sensor component of the inflammasome for a diversity of inflammatory stimuli, has also been implicated in the progression of PD. Fibrillar α‐synuclein activates microglial NLRP3 resulting in neuroinflammation and toxicity to dopaminergic neurons (Gordon et al., 2018). Interestingly, deletion of autophagy‐specific ATG5 from microglia results in neuroinflammation, reduction in dopaminergic neurons, and motor impairments dependent on NLRP3 inflammasome activation (Cheng et al., 2020). Given this, α‐synuclein may activate NLRP3 in microglia by way of protein aggregation affecting autophagic processes. These results present a potential mechanism by which neuroinflammation produced by activated microglia may be responsible for neuronal loss in PD.

#### 5.1.3 Altered astrocyte activity

In addition to microglia, astrocytes can also contribute to the progression of AD through their transformation into reactive astrocytes. It has been reported that these astrocytes lose their capacity to promote neuronal survival and synaptogenesis and can contribute to neuronal death (Liddelow et al., 2017). Moreover, the interaction between astrocytes and Aβ has been implicated in the negative impact of glial dysfunction on neuronal viability. Allaman and colleagues, using astrocyte‐neuron co‐cultures, showed that global changes in astrocyte metabolism caused by Aβ compromise neuronal viability (Allaman et al., 2010). Similarly, Paradisi and colleagues reported that astrocytes can shield neurons from Aβ‐induced neurotoxicity; however, this protective function is diminished upon interaction with Aβ, resulting in increased neurotoxicity (Paradisi et al., 2004). The in vitro knockdown of astrocytic TGF‐β1, a regulator of synapses, showed an exacerbation of the Aβ‐mediated synaptotoxic effect because of the decreased ability of astrocytes to protect synapses (Diniz et al., 2017). Another study, utilizing induced pluripotent stem cells (iPSCs) derived from AD patients carrying Presenilin1 mutations, revealed common features of the disease pathology, particularly within astrocytes. Noteworthy findings included an elevation in Aβ production, altered cytokine release such as IL‐2, IL‐6, IL‐10, and granulocyte‐macrophage colony‐stimulating factor (GM‐CSF), and alterations in energetic metabolism characterized by increased oxidative stress and diminished lactate secretion (Oksanen et al., 2017). Additionally, co‐cultures of healthy neurons with AD astrocytes revealed dysregulation of Ca2+ homeostasis and substantial changes in neuronal firing patterns, suggesting a diversity of mechanisms through which astrocytes may contribute to AD pathology (Oksanen et al., 2017).

Similarly, there has recently been a greater appreciation for the role astrocytes may play in PD progression. Considering that neurotoxic astrocytes contribute to dopaminergic neuronal death and produce motor deficits in animal models of PD, an understanding of how astrocytes become reactive may provide therapeutic opportunities before neurons become permanently lost. Several lines of evidence suggest that PD mutations result in transient or long‐term alterations to astrocytes that promote reactivity and toxicity. For example, astrocytes derived from iPSCs from patients with LRRK2‐G2019S mutations have increased α‐synuclein expression and displayed increased cytokine release under inflammatory conditions (Sonninen et al., 2020). Furthermore, LRRK2‐G2019S mutant astrocytes show a decreased ability to degrade α‐synuclein via the endolysosomal pathway (Streubel‐Gallasch et al., 2021). Indeed, it has been shown that dopaminergic neurons cultured in the presence of LRRK2‐G2019s mutant astrocytes are more susceptible to neuronal loss, which reinforces the role of astrocytic LRRK2 mutations in neurodegeneration (di Domenico et al., 2019). Taken together, astrocytes may play a role in non‐cell autonomous neuronal loss in PD, and strategies to retain normal astrocyte physiology may be beneficial when designing potential treatment strategies.

### 5.2 Stress‐related neuropsychiatric disease: PTSD, anxiety, schizophrenia, and depression

The pathophysiology of psychiatric disorders has been associated with neuronal damage involving changes in synaptic function and neurotransmission imbalance in specific brain circuits. Unlike what is observed in neurodegenerative diseases, psychiatric diseases do not have prominent characteristics of neuronal loss (reviewed by (Duman et al., 2016; Lewis & Sweet, 2009). Recently, there has been a greater appreciation for the involvement of inflammation and immune dysregulation in the pathophysiology of psychiatric disorders. Inflammatory processes in the brain have been implicated in conditions such as depression, schizophrenia, and bipolar disorder (Köhler et al., 2014). Many psychopathologies share stress as a common risk factor; exposure to stressful events may increase considerably during adolescence, a vulnerable period to the negative effects of stress (reviewed by (Callaghan & Tottenham, 2016; Nelson & Gabard‐Durnam, 2020)). When such stress is perceived, it triggers cascades of endocrine, immune, and neural responses that include the release of cortisol and pro‐inflammatory cytokines (reviewed by (Nusslock & Miller, 2016)) and epigenetic changes (Francis et al., 1999; Meaney & Szyf, 2005). These mechanisms may trigger neuroplasticity, allowing the brain to adapt and deal with the challenges presented by the new environmental context and potential future threats (McEwen, 2004). However, when the mechanisms are not well‐controlled, they may lead to excess inflammation and immune dysregulation contributing to psychiatric disorders.

In stress‐related neuropsychiatric disorders, many structural alterations in the brain have been linked to inflammation via activation of microglia and/or astrocytic dysfunction (Figure 4a,b). A range of psychosocial stressors, from early‐life/prenatal stress to stress during adulthood, promote the increase of microglial activity in the hippocampus (reviewed by (Calcia et al., 2016)). Specifically, heightened inflammation, which affects synaptic plasticity (Riazi et al., 2015), is present in major depressive disorder (MDD) (Bai et al., 2020) and post‐traumatic stress disorder (PTSD). Given that stress‐related neuropsychiatric disorders are often associated with changes in brain/gray matter volume, synaptic connections, and synaptic plasticity (Ansell et al., 2012; Etkin & Wager, 2007; Holmes et al., 2019; Kassem et al., 2013; Papagni et al., 2011; Shin et al., 2006), the understanding of how inflammation, including complement and cytokine signaling, and glial dysfunctions contribute to such changes may lead to new mechanistic insights and therapeutic avenues for neuropsychiatric disorders.

#### 5.2.1 Complement signaling

The complement system that mediates neurodevelopmental synaptic pruning has been linked to synapse loss observed in pathological brains (reviewed in (Druart & Le Magueresse, 2019)). The typical manifestation of schizophrenia symptoms in late adolescence or early adulthood aligned with the emergence of the Feinberg synaptic pruning hypothesis that postulates a defective maturational process related to aberrant synaptic pruning as a potential underlying cause of the disease (Feinberg, 1982). Neuroimaging studies in schizophrenia (Honea et al., 2005; Sweet et al., 2009) and bipolar disorder patients (Hibar et al., 2018) revealed increased cortical thinning, that possibly reflects excessive pruning. The complement component 4 (C4A) has been linked to complement‐mediated synaptic pruning and cortical thinning in schizophrenia (Sekar et al., 2016). Phosphorus magnetic resonance spectroscopy in adult‐onset schizophrenia patients with high C4A gene copy numbers detected increased neuropil contraction in the prefrontal and parietal regions, whereas adolescent‐onset patients with high C4A gene copy numbers showed increased neuropil contraction in the prefrontal cortex and thalamus (Prasad et al., 2018). In MDD patients, there is synaptic loss and connectivity dysfunction (Holmes et al., 2019), and the significantly higher concentration of C3 and C3a in the peripheral plasma of medication‐free MDD groups suggest that complement signaling may also be implicated in the pathophysiology of MDD (Luo et al., 2022).

#### 5.2.2 Activated microglia and pro‐inflammatory conditions

The impact of neuroinflammation on neuroplasticity has also emerged as a widely studied mechanism in relation to the pathogenesis of neuropsychiatric diseases (reviewed by (Chagas et al., 2020)). Indeed, several neuropsychiatric diseases are marked by higher concentrations of pro‐inflammatory cytokines. More specifically, patients with PTSD demonstrate increased levels of TNF‐α, IL‐1β, IL‐6, and C‐reactive protein (CRP) (Guo et al., 2012; Hoge et al., 2009; Tursich et al., 2014). Similarly, in both first episode and relapsed cases of schizophrenia there is an increase in pro‐inflammatory cytokines (Goldsmith et al., 2016; Miller et al., 2011). Further strengthening the idea of inflammation in neuropsychiatric conditions are findings that patient cohorts of MDD and bipolar disorder also have increased levels of pro‐inflammatory cytokines (Köhler et al., 2017; Modabbernia et al., 2013).

There are several lines of evidence linking inflammation to neuropsychiatric disorders using lipopolysaccharide (LPS; a component of gram‐negative bacteria) as a robust activator of microglia and neuroinflammation. Using an LPS‐induced inflammation model, Cao and colleagues demonstrated that early‐stage inflammation increases the risk of developing depression during adolescence through the dysregulation of microglial capacity to engulf neuronal spines. This dysregulation led to long‐lasting maladaptation of glutamatergic neurons in the anterior cingulate cortex to stress, ultimately contributing to the development of depression‐like symptoms in adolescence (Cao et al., 2021). Furthermore, LPS‐induced dysfunction of neural plasticity in the amygdala has also been linked to anxious and depressive‐like behaviors. Recently, Zheng and colleagues observed microglia activation and the release of pro‐inflammatory cytokines in the basolateral amygdala (BLA), along with an imbalance between excitatory and inhibitory neurotransmission after LPS‐induced neuroinflammation (Zheng et al., 2021).

In addition, the dysfunction of glial cells in schizophrenia patients seems to play an important role in disease pathology. For instance, it has been demonstrated that microglia‐like cells from schizophrenia patients display increased synapse engulfment, which may be partly mediated by a genetic schizophrenia‐risk variant (Sellgren et al., 2019). The intricate relationship between neuroinflammation and neuroplasticity in the pathogenesis of neuropsychiatric diseases may offer potential avenues for therapeutic intervention targeting neuroinflammatory processes and neural plasticity.

#### 5.2.3 Altered astrocyte activity

The role of astrocytes in stress‐related psychiatric disorders has also received significant attention in recent years, considering their roles in different stages of development and in homeostasis (see Sections 2 and 3; reviewed by (Rajkowska, 2000; Rajkowska & Miguel‐Hidalgo, 2007; Yamamuro et al., 2015)). Recently, Byun and colleagues demonstrated in a model of early social deprivation and using brain organoids, that stress hormones enhance astrocyte‐mediated phagocytosis of excitatory synapses through the increase of MERTK. In this work, the authors correlate this mechanism to abnormal networks, which could result in the complex behavior observed in mental health conditions such as social deficiencies and depression originating from childhood neglect and/or abuse (Byun et al., 2023). There is also accumulating evidence implicating astrocytes in the pathogenesis of PTSD, with studies highlighting their involvement in the aberrant formation and remodeling of fear memories and stress‐related dysfunctions. The reduction of Ca2+ activity in astrocytes is followed by increased fear memory and dysregulation of the anxiolytic effect mediated via adenosine A1 receptor activation (Li et al., 2020b). Experimental models have also reported decreased levels of astrocyte‐related proteins, such as GFAP, and atrophic astrocytes in corticolimbic brain areas as being implicated in PTSD (Ongür et al., 1998; Saur et al., 2016). Another study utilizing PET with the monoamine oxidase B (MAO‐B) radioligand [11C]SL25.1188 suggested a potential loss of astrocytes or independent downregulation of MAO‐B in individuals with PTSD, particularly those with more severe negative affect (Gill et al., 2022). These findings align with preclinical literature and recent observations of decreased [11C]PBR28 PET brain imaging of TSPO, a widely used biomarker of neuroinflammation (Bhatt et al., 2020). Also, in mouse models of anxiety, there is a correlation between astrocytic activity and affective states, demonstrated through in vivo astrocytic calcium imaging in the hippocampus (Cho et al., 2022).

In summary, stress response is a crucial physiological and homeostatic response that can become chronic and maladaptive in the face of adversity, and it can contribute to the appearance of many psychiatric disorders. Ongoing research aims to unravel the complex relationships behind the significant role that the environment plays in shaping neurodevelopmental processes, how it influences mental health outcomes, and how the rescuing of neurodevelopmental programs, especially those involving glial cells (as described here), can potentially support new avenues for intervention, prevention or promotion of positive mental health outcomes in individuals facing adversity.

### 5.3 Demyelinating diseases: multiple sclerosis

In the adult brain, demyelination in the CNS can result from an injury or stroke, genetic mutation, or autoimmune origin. One of the most extensively researched demyelinating diseases is multiple sclerosis (MS). It is characterized by periods of neuroinflammation that cause myelin degradation in both the gray and white matter of the CNS. This leads to progressive neuronal loss because of damage of myelin sheaths wrapping the axons, which results in cognitive impairment in chronic stages (reviewed by (Inglese & Petracca, 2015; Nave, 2010). Axonal and neuronal damage is a major contributor to the progressive nature of chronic demyelinating diseases, such as that occurring in MS (reviewed by (Trapp & Nave, 2008)). Therefore, an understanding of the mechanisms that govern demyelination and those that prevent remyelination is necessary for the development of therapeutics (Figure 4c).

Age‐related impairments in OPC differentiation are a major factor in remyelination failure because they affect spontaneous remyelination and eventually cause axon degeneration (reviewed by (Leenders et al., 2024; Tepavčević & Lubetzki, 2022)). During chronic demyelinating episodes, OPC pools become depleted and remyelination is impaired because of the lack of OPC availability (Armstrong et al., 2006; Mason et al., 2004). Supporting the idea of diminished OPC availability are findings demonstrating that MS patients have OPC‐expressed antigens (NG2)‐recognizing antibodies (Niehaus et al., 2000). Furthermore, alterations in the local expression of the OPC migration guidance cues, Semaphorin 3A and 3F, may also contribute to the failure of OPC recruitment to areas of demyelination (Williams et al., 2007). Additionally, remyelination may fail because OPCs become impaired in their maturation and differentiation, as several markers of oligodendrocytes such as O4, NG2, PLP, Olig2, and Nkx2.2 are affected during chronic stages of MS (Chang et al., 2002; Kuhlmann et al., 2008; Wolswijk, 1998).

Astrocytes and microglia have also been shown to play a role in myelination. Lessons from development have shown that microglia play a role in the phagocytosis of OPCs. More specifically, myelination is in part regulated by fractalkine‐receptor‐mediated phagocytosis, as this mechanism is involved in homeostatic myelination in early postnatal development (Nemes‐Baran et al., 2020). After development, microglia continue to have pro‐myelinating effects in the adult brain (reviewed by (Lloyd & Miron, 2019)). With respect to astrocytes, they are required for oligodendrocyte differentiation, and activated astrocytes promote myelination (Meyer‐Franke et al., 1999; Nash et al., 2011). There is a report that overexpression of GFAP prevents demyelination induced by a cuprizone diet in rodents (Kramann et al., 2019). Mechanistically, astrocytes may promote remyelination via an increase in CNTF, which stimulates the production of FGF‐2 that regulates the mitosis of OPCs (Albrecht et al., 2003). In addition, astrocytes may also increase OPCs via the secretion of osteopontin (Opn) (Selvaraju et al., 2004).

However, in demyelinating diseases such as MS, glial cells withdraw their homeostatic features and adopt a pro‐inflammatory/toxic phenotype (reviewed by (Yong, 2022)). In MS, the microglial transcriptome is altered, and there is a decrease in homeostatic microglia and an increase in activated microglia (Böttcher et al., 2020; Masuda et al., 2019) that are toxic to oligodendrocytes (Liddelow et al., 2017). Consistent with this, microglia activation during methotrexate chemotherapy depletes white matter OPCs and results in myelination deficits (Gibson et al., 2019). It is also worth noting that hypertrophic reactive astrocytes can produce a glial scar in experimental autoimmune encephalomyelitis (EAE) models of demyelination, which is sufficient to impair oligodendrocyte migration to the lesion sites (Bannerman et al., 2007). Furthermore, in the same model, the blockade of the neurovascular damage induced by astrocytes was sufficient to increase myelin basic protein (MBP) and improve neurological outcomes (Eilam et al., 2018). Finally, the release of pro‐inflammatory cytokines from glia such as INFγ may result in STING activation within neurons which can further amplify glia‐mediated damage (Woo et al., 2024). Taken together, astrocytes and microglia can influence the extent of myelination completed by oligodendrocytes. Although these processes are tightly regulated during development and homeostasis, there is dysregulation during demyelinating conditions in which astrocytes and microglia negatively impact the health of white matter.

## 6 TARGETING GLIAL CELLS TO REBUILD THE NERVOUS SYSTEM

### 6.1 Targeting glia to prevent neuronal degeneration and dysfunction

**FIGURE 5:**
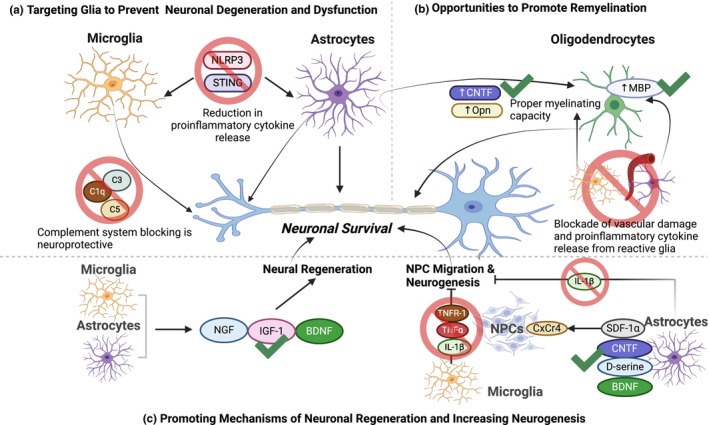
Targeting glial cells to rebuild the nervous system. (a) Blocking microglia or astrocyte conversion to non‐physiological phenotype offers neuronal protection through inhibiting pro‐inflammatory cytokine release (NLRP3, STING) and reducing inappropriate complement‐mediated (C3, C1q, C5) phagocytosis of synapses. (b) Targeting oligodendrocytes and their interactions with reactive glial cells promotes increases in myelination. A reduction in vascular damage or pro‐inflammatory cytokines secretion associated with reactive glia can lead to increases in pro‐myelination signals including MBP, CNTF, and Opn. (c) Modulation of glial cell activities demonstrates the potential to enhance neuronal regeneration and improve NPC migration and neurogenesis. Targeting microglia or astrocytes to stimulate the release of trophic factors such as NGF, IGF‐1, and BDNF may promote neuronal regeneration. Increasing signaling molecules from astrocytes such as SDF1α, CNTF, D‐serine, and BDNF may improve neurogenesis. Neuronal regeneration and neurogenesis may also be enhanced through a reduction of pro‐inflammatory molecules such as IL‐1β and TNFα released from microglia and astrocytes. Created with Biorender.com.

As described above, microglia, astrocytes, and oligodendrocytes play a critical role in brain development and function (Figures 1, 2). However, in pathophysiological conditions such as AD, PD, MS, and neuropsychiatric conditions these cells may withdraw their supportive functions and transition towards an aberrant phenotype which promotes toxicity and dysfunction of neurons (Figure 4). In this section, we describe how targeting glial cells represents a therapeutic strategy that may be beneficial across a broad group of neurodegenerative and neuropsychiatric conditions. We believe targeting glial cells will allow for (1) prevention of non‐cell autonomous degeneration and dysfunction, (2) opportunities to promote remyelination, and (3) promote neuronal regeneration and neurogenesis (Figure 5).

Glial cells play a role in neuronal dysfunction associated with neurodegenerative and neuropsychiatric disease (Section 5 and Figure 4). Targeting microglia or astrocytes may prevent non‐cell autonomous neuronal/synapse loss (Figure 5a). Indeed, targeting the complement pathway in neurodegenerative diseases may prevent synapse loss. Studies have shown that C3 deletion from the APP/PS1 mouse model of AD protected against age‐dependent synapse loss and neuronal death (Shi et al., 2017). Furthermore, these animals showed a reduced level of deficits in behavioral tasks associated with memory and learning (Shi et al., 2017). In addition, APP/PS1 animals lacking C1q showed decreased levels of glial activation and a reduction in hippocampal neuron loss (Fonseca et al., 2004). Consistent with this, complement knockout in AD mouse models showed rescued synapse and neuronal loss, resulting in improvements in neuronal function and behavioral outcomes (Wu et al., 2019). In PD models, pharmacological inhibition of C5 and C3 complement proteins was sufficient to block α‐synuclein‐induced cell death in vitro (Gregersen et al., 2021). Furthermore, Zhang and colleagues demonstrated that microglia‐specific delivery of a complement inhibitor protects against dopaminergic neuron loss and rescues behavioral deficits associated with a mouse model of α‐synuclein aggregation in vivo (Zhang et al., 2023b). Similar to AD and PD, neuropsychiatric conditions may result from dysregulation of the complement pathway (see Section 5). Interestingly, complement C3 knockout mice demonstrate differences in synaptic pruning and are more resilient to chronic stress and depressive‐like behaviors (Crider et al., 2018; Wang et al., 2023). These examples show that targeting complement signaling in neurodegenerative and neuropsychiatric diseases may be a valuable therapeutic approach to suppress the aberrant loss of synapses and protect vulnerable neuronal populations.

Periods of neuroinflammation in neurodegenerative and neuropsychiatric diseases that precede neuronal dysfunction represents an opportunity in which targeting aberrant glial activity may offer protection prior to neuronal loss. In AD models, modulating pro‐inflammatory glia activity has been shown to be beneficial in reducing disease pathology and improving behavior in several animal models. More specifically, mutant Tau mice display increased immune responses, and depletion of microglia was sufficient to block Tau‐induced neurodegeneration (Chen et al., 2023). Moreover, microglia‐specific deletion of an AD risk gene, APOE4, resulted in decreased pathology and increased neuroprotection in both amyloid and tau mouse models (Yin et al., 2023). Furthermore, pharmacological manipulation of microglia via a glucagon‐like peptide 1 (GLP1) attenuated pro‐inflammatory cytokine release, blocked the conversion of astrocytes to a reactive state, preserved neuronal viability, and improved learning and memory in an AD mouse model (Park et al., 2021).

In animal models of PD, reducing reactivity in microglia and astrocytes has been shown to protect dopaminergic neurons. In an MPTP model of PD, the blockade of microglial reactivity with minocycline prevented dopaminergic neuron loss and rescued striatal dopamine depletion (Wu et al., 2002). Furthermore, α‐synuclein has been shown to cause pro‐inflammatory cytokine release via STING and, accordingly, researchers have demonstrated that STING‐deficient mice injected with α‐synuclein preformed fibrils are resistant to dopaminergic neuron loss and motor deficits (Hinkle et al., 2022). In addition, small‐molecule inhibitors of NLRP3 block inflammasome activation in microglia and are sufficient to rescue dopaminergic neuron loss and motor deficits in a synucleinopathy model (Gordon et al., 2018). Aside from microglia, targeting astrocytes may also be beneficial in PD. Administration of a pharmacological necroptosis inhibitor has shown efficacy in reducing astrocyte reactivity and attenuating the loss of dopaminergic neurons and subsequent behavioral deficits in an MPTP model of disease (Qiao et al., 2023). However, it should be noted that protection of neurons and reduction of astrocyte reactivity reported in this study could be because of the inhibitor acting directly on neurons. Recent findings have suggested that astrocytes exert their pro‐death effects on neurons via secreting toxic lipids (Guttenplan et al., 2021). In a PD model, manipulation of astrocytes via AAV‐mediated expression of transmembrane protein 164 (TMEM164), a protein thought to be involved in ferroptosis, resulted in a reduction in the amount of neurotoxic saturated lipid release and prevented the loss of dopaminergic neurons and motor deficits (Zhang et al., 2023a). Thus, targeting both microglia and astrocytes seems to be a beneficial strategy to reduce neuronal loss in PD models. Another strategy may be to target microglia to prevent the activation of astrocytes and decrease neuronal death. Indeed, Yun and colleagues showed that a GLP‐1R agonist blocks the microglia‐mediated conversion of astrocytes to neurotoxic reactive astrocytes and attenuates dopaminergic neuron loss and behavioral deficits in both mice injected with preformed α‐synuclein fibrils and those harboring an A53T α‐synuclein mutation (Yun et al., 2018).

Reducing microglial activation and blocking the conversion of astrocytes to a toxic state may also improve various neuropsychiatric conditions. More specifically, microglia‐specific deletion of the key inflammasome regulator NLRP3 attenuates neurotoxic astrocytes and block adverse behaviors in mice with a depressive‐like phenotype (Li et al., 2022). Pharmacological blockade of glial cell reactivity may also be a viable strategy in neuropsychiatric models, as it has been shown that fluoxetine, a commonly used antidepressant, blocks the accumulation of toxic astrocytes and attenuates depressive behavior in mice subjected to chronic mild stress (Fang et al., 2022). Similarly, chronic pre‐treatment with fluoxetine significantly prevented the neurophysiological changes induced by LPS and alleviated anxiety and depressive‐like behaviors (Zheng et al., 2021). This suggests that fluoxetine may exert its therapeutic effects, at least in part, by mitigating the detrimental effects of neuroinflammation exerted by glial cells. Furthermore, activating astrocyte function with optogenetic activation reduced anxiety‐like behavior and increased excitatory synaptic transmission (Cho et al., 2022). Together, these results suggest that blocking glial reactivity and transitioning microglia and astrocytes toward a more homeostatic state may be potential therapeutic strategies in treating neurodegenerative and neuropsychiatric conditions.

### 6.2 Opportunities to promote remyelination

Remyelination is a process that occurs in an attempt to repair damaged myelin (Figure 5b). This regenerative process is driven by adult multipotent OPCs that proliferate and migrate from their niches to damaged sites, differentiating into mature oligodendrocytes that produce new myelin sheaths (reviewed by (Dimou & Gallo, 2015; Franklin & Ffrench‐Constant, 2017)). Recently, it has been shown that parenchymal demyelinated oligodendrocytes that survived the lesion are also capable of forming new myelin sheaths, therefore contributing to repair (Bacmeister et al., 2020; Duncan et al., 2018). Furthermore, after an acute demyelinating episode, the density of OPCs tends to increase (Levine & Reynolds, 1999), resulting in increased migration towards the lesion sites (reviewed by (Franklin & Blakemore, 1997)). Under these demyelinating circumstances, OPCs become activated and release cytokines IL‐1 and CCL2, which encourage OPC mobilization and repopulation to demyelinated regions (Moyon et al., 2015). OPC density gradually returns to normal levels as mature oligodendrocytes reappear in the lesion. Using transgenic animals such as PDGFRaCreERT2:RosaYFP and NG2CreERT2:TaumGFP mice, OPCs and their offspring were traced after tamoxifen injection, and this provided spatiotemporal evidence for the production of remyelinating oligodendrocytes from OPCs (Mei et al., 2016; Zawadzka et al., 2010). These results suggest that the parenchymal OPC pool is an initiator of remyelination, which is followed by OPC populations that migrate and remyelinate lesion sites.

Unfortunately, remyelination efficiency declines with age (Neumann et al., 2019; Sim et al., 2002), representing a barrier in therapies targeting diseases such as MS. Therefore, an understanding of why remyelination fails in adults may uncover potential strategies to overcome the remyelination barrier. Microglia and astrocytes may contribute to the ability of oligodendrocytes to remyelinate following a lesion. On one hand, Gibson and colleagues showed that the depletion of microglia results in normalization of OPC behavior, increased myelination, and rescue of cognitive behavior after methotrexate chemotherapy (Gibson et al., 2019). This study suggests that reversing activated pro‐inflammatory microglia may be a beneficial strategy to promote oligodendrocyte mediated myelination. Additionally, microglia can facilitate repair of demyelinated lesions through the upregulation of cholesterol precursors to resolve inflammation and promote oligodendrocyte differentiation and remyelination (Berghoff et al., 2021). On the other hand, astrocytes may also be targeted to promote oligodendrocyte differentiation and improve myelination outcomes. Aged astrocytes have been shown to withdraw myelinating support, with rapamycin, a mTOR inhibitor, being able to reverse these effects and support oligodendrocyte differentiation (Willis et al., 2020). Furthermore, astrocytic mGluR5 may be targeted to improve myelination and behavioral outcomes via BDNF secretion in a cuprizone diet model of demyelination (Saitta et al., 2021). Interestingly, microglia and astrocytes have been shown to participate in brain remyelination by expressing Opn and treatment with Opn was shown to be effective at increasing both MBP and myelin sheath formation in in vitro models of myelination (Selvaraju et al., 2004). In summary, targeting microglia and astrocytes represents a reasonable approach to promote increased myelination of the nervous system.

### 6.3 Promoting mechanisms of neuronal regeneration and increasing adult neurogenesis

#### 6.3.1 Axonal outgrowth and neuronal migration

In addition to preventing non‐cell autonomous neurodegeneration and strategies to increase myelination, improving the processes of axonal outgrowth and neuronal migration, together with adult neurogenesis, are integral to neural repair (Figure 5c). Axonal regeneration, the complex process involving the regrowth of damaged axons, plays a critical role in neural repair (Mahar & Cavalli, 2018; Winter et al., 2022). In adulthood, this process is often hindered by the inhibitory environment of the CNS, particularly the extracellular matrix and the formation of glial scars, and the poor regenerative ability of mature neurons (reviewed by (Fawcett, 2020)). The success of axonal regeneration is influenced by the fine balance between growth‐promoting and growth‐inhibiting factors, as well as the coordinated actions of specific intracellular signaling mechanisms, transcription factors and epigenetic modifiers that modulate this process (Liu et al., 2011).

Several neurotrophic factors, including BDNF, IGF‐1, and nerve growth factor (NGF), have been identified as key drivers of cytoskeletal reorganization and axonal outgrowth (reviewed by (Lykissas et al., 2007)). As seen in development, neurotrophin gradients guide axons to their target sites, and astrocytes and radial glia function as scaffolding guiders (reviewed by (Huang & Reichardt, 2001)). Therefore, ensuring sufficient levels of these molecules for axons and providing permanent glial support would be powerful stimuli for regeneration. For instance, implantation of BDNF‐hypersecreting mesenchymal stem cells after spinal cord injury stimulated the regeneration of spinal axons and improved functional recovery (Sasaki et al., 2009). More recently, in adult mice, co‐overexpression of IGF‐1/Opn/CNTF was found to induce the regrowth of retinal axons and the formation of functional synapses in the superior colliculus (Bei et al., 2016). However, it should be noted that regenerated axons were not myelinated, and functional improvements only occurred in the presence of a pharmacological agent that improved conduction.

An alternative to safeguarding the availability of trophic factors or activating their receptors is to intervene directly in the corresponding signaling pathway. Manipulating signaling pathways related to neuronal growth invigorates axon regeneration and positively influences functional outcomes. Several studies have identified the PI3K/Akt/mTOR and JAK/STAT signaling pathways as key regulators of the potential of CNS nerve cells to regenerate. For example, co‐deletion of PTEN, a negative regulator of the mTOR pathway, and SOCS3, a negative regulator of JAK/STAT signaling, was found to enable long‐distance axon regeneration in adult retinal ganglion cells (RGCs) following a nerve crush injury (Sun et al., 2011). Similarly, inhibition of RhoA, a small GTPase protein of the Rho family, in neurons was shown to reverse actin compacting through myosin II and enable microtubule protrusion in the axon tip, thus, facilitating axon regeneration (Stern et al., 2021). These observations support the narrative that at least some of the mechanisms that regulate axon outgrowth during development are recapitulated during regeneration, further emphasizing the importance of utilizing these processes to accelerate CNS regeneration upon injury or system failure.

Neuronal migration is also a critical process during development that holds significant potential for CNS regeneration in adulthood (reviewed by (Lui et al., 2011)). During development, radial glial cells serve as scaffolds for migrating neurons. Through reelin signaling, neurons often migrate along radial glial fibers, which provide physical support and guidance (reviewed by (Götz & Huttner, 2005)). In adulthood, these glial cells could be manipulated to act as guides for the migration of newly generated neurons to their desired locations for regeneration. Molecules such as netrins, slits, semaphorins, ephrins and chemokines provide repulsive and attractive guidance signals that steer migrating neurons in the right direction (reviewed by (O'Donnell et al., 2009)) and may be potentially utilized in the compromised CNS to direct newly generated neurons to specific areas of the brain for targeted regeneration. Further reinforcing this, cell adhesion molecules present during development, including L1 and neural cell adhesion molecule (NCAM)—the latter being also present during adulthood—could be used to influence de novo formation of neuronal processes and facilitate the successful migration of newborn cells to target sites (Jakovcevski et al., 2013; Kanemaru et al., 2013). On the other hand, and importantly, a fine balance between disposing of local obstructive components (e.g., reactive glia) and enabling the migration of protective elements (e.g., nutrients and scaffolding proteins) is essential to accelerate regeneration (Anderson et al., 2016; Duraikannu et al., 2019; Faiz et al., 2015).

To facilitate intrinsic cues for neuron migration, one could promote their activities through the manipulation of microglia and astrocytes. Imitola and colleagues demonstrated that astrocytes and endothelial cells upregulate the inflammatory chemoattractant stromal cell‐derived factor 1α (SDF‐1α), which stimulates the cognate receptor CXCR4 expressed on NSCs, promoting their proliferation and migration towards infarct regions (Imitola et al., 2004). Other studies further supported that SDF‐1α/CXCR4 enhances the mobilization and homing of exogenously transplanted NSCs to injury sites in mouse brains (Xue et al., 2014; Zhang et al., 2013). In addition, microglia may stimulate axon growth and could be exploited towards a strategy to improve neuronal regeneration. More specifically, microglial activation supports axonal growth, presynaptic differentiation, and neuroplasticity in lesion models (Chagas et al., 2019; Jiang et al., 2019). Furthermore, microglia deficient in fractalkine receptor Cx3cr1 promote the repair of serotonergic neurons in a spinal cord injury model (Freria et al., 2017). Taken together, these results point to therapeutic strategies in which focusing on glial cells in addition to regulating the intrinsic properties of neurons could enhance neuronal regeneration.

#### 6.3.2 Increasing adult neurogenesis

Increasing adult neurogenesis offers a potential therapeutic strategy for neurodegenerative diseases and neuropsychiatric disorders. Evidence suggests that neurogenesis is required for memory, learning, and emotional regulation (Clelland et al., 2009; Sahay et al., 2011; Santarelli et al., 2003). Enhancing adult neurogenesis can counter the age‐related decline in cognitive function and this holds the potential to reduce the impact of neurodegenerative diseases such as AD (Shors et al., 2001). AD involves the progressive loss of cognitive abilities with age partly because of the reduction of adult neurogenesis (Mu & Gage, 2011; Vecchio et al., 2018). Strategies aiming to normalize neurogenesis as potential therapeutic approaches to the condition are yet to be confirmed. One challenge is to ensure that interventions promote the formation of functionally appropriate neural networks without exacerbating abnormal brain activities, such as seizure activity.

Growth factors, cytokines, transcription factors, cell–cell adhesion molecules and epigenetic factors have been identified as stimulators of adult neurogenesis (reviewed by Hagg, 2009; Hodge & Hevner, 2011; Vilar & Mira, 2016)). These factors drive fate specification and proliferation of NSCs and support the survival and integration of newly generated neurons into existing neural circuits (reviewed by (Cotman et al., 2007)). Through the induction of specific cellular processes and directed transcriptional programs (details in (Ninkovic & Götz, 2007), neural stem and progenitor cells can be harnessed to ameliorate brain damage, counteract neurodegeneration, and alleviate symptoms in neuropsychiatric conditions (Faiz et al., 2015; Nie et al., 2023; Rodrigues et al., 2020). However, switching the expression levels of these factors requires controlled and efficient manipulation to avoid undesired effects (Obernier et al., 2018). Strategies aimed at potentiating molecular mechanisms that protect nerve cells against damage and propel intrinsic regeneration (e.g., combinatory approaches) would be beneficial for accelerating NSC division and differentiation in the context of injury or pathology (DePaul et al., 2017). Recently, glia have been shown to influence neurogenesis (see Section 1.2.4). Specifically, astrocytes and microglia can influence the survival and proliferation of precursor cells and ultimately influence their commitment to a neuronal phenotype (Song et al., 2002). Given this, strategies that regulate astrocytic and microglial function may be beneficial in promoting neurogenesis in a variety of brain disorders. Indeed, activation of proinflammatory cytokine IL‐1β, which can be released by microglia or astrocytes, is sufficient to suppress neurogenesis and either pharmacological blockade or deletion of IL‐1β receptor rescues neurogenesis in mouse models of diseases (Gemma et al., 2007; Ja & Duman, 2008; Wu et al., 2012). Studies on minocycline, an anti‐inflammatory drug, revealed its potential in restoring hippocampal neurogenesis by inhibiting microglial reactivity (Ekdahl et al., 2003). Additionally, systemic non‐steroidal anti‐inflammatory drug (NSAID) indomethacin blocked the detrimental effects of peripheral LPS on neurogenesis by reducing activated microglia and increasing the number of newly born neurons in rats (Monje et al., 2003). In another study, the numbers of proliferating and mature neuronal cells in the dentate gyrus increased after indomethacin administration 1 day prior to brain injury induction via photothrombosis, suggesting its potential to enhance neurogenesis after brain injury (Kluska et al., 2005). Clinical relevance was also observed in a cohort study evaluating the potential of indomethacin and other NSAIDs in ameliorating memory loss in AD patients (in ‘t Veld et al., 2001), possibly attributed to neurogenesis promotion by microglial inhibition. Chronic treatment with doxycycline, an antibiotic derivative with anti‐inflammatory properties, also increases neurogenesis, with new neurons displaying increased spine density (Sultan et al., 2013). Consistent with this, doxycycline may promote neurogenesis in PD models as it has been proposed as a therapeutic approach in preclinical studies of PD and L‐DOPA‐induced dyskinesia (Del‐Bel et al., 2023; dos Santos et al., 2022; Santos‐Lobato et al., 2023).

Taken together, potential therapeutic applications of promoting adult neurogenesis by targeting glia hold promise for a range of neurological and psychiatric conditions. However, the complexity of neurogenesis and its regulation in the adult brain presents significant challenges. It is critical to understand the glial mechanisms that govern neurogenesis and how they can be manipulated to repair and regenerate neural circuits in various pathological conditions. Future strategies may focus on complementary approaches that enhance the intrinsic properties of neurons and the neurogenesis‐promoting functions of glia that improve the functional integration of newborn cells into existing circuitry. Animal studies are promising, and the translation of these findings in humans should follow.

## 7 CONCLUSION

The intricate interplay between glia and neurons underscores the dynamic nature of the nervous system, spanning from development to disease. Throughout this review, we have explored the multifaceted roles of glia in shaping neural circuitry, maintaining homeostasis, and influencing neuronal vulnerability. From providing structural support and trophic factors during development to modulating synaptic transmission and neurotransmitter recycling in adulthood, glial cells play an indispensable role in ensuring the proper function of neuronal networks. Moreover, the vulnerability of neurons to various insults, including oxidative stress, inflammation, and excitotoxicity, highlights the pivotal role of glia in safeguarding neuronal health. Dysregulation of glial functions has been implicated in numerous neurodegenerative and neuropsychiatric diseases, where glial dysfunction exacerbates neuronal loss and contributes to disease progression.

Accordingly, emerging evidence suggests that targeting glial cells should hold promise as a therapeutic strategy for ameliorating dysfunctions in neurodegenerative and neuropsychiatric diseases. By promoting physiological glial activity and attenuating non‐physiological aberrant activity, novel therapeutic interventions should aim to mitigate neuroinflammation, promote neuroprotection, and enhance neural repair processes. Understanding the intricate crosstalk between glia and neurons provides valuable insights into the pathophysiology of neurological and neuropsychiatric disorders and unveils new avenues for therapeutic intervention. Future research endeavors focused on elucidating the molecular mechanisms underlying glia–neuron interactions will undoubtedly pave the way for innovative treatments aimed at restoring neuronal health and function in neurological diseases and rebuilding the nervous system.

## AUTHOR CONTRIBUTIONS

**Matthew D. Demmings:** Conceptualization; visualization; project administration; writing – original draft; writing – review and editing. **Luana da Silva Chagas:** Conceptualization; writing – original draft; writing – review and editing. **Marianela E. Traetta:** Conceptualization; writing – original draft; writing – review and editing. **Rui S. Rodrigues:** Conceptualization; writing – review and editing; writing – original draft. **Maria Florencia Acutain:** Writing – original draft. **Evgeny Barykin:** Writing – original draft. **Ashok Kumar Datusalia:** Writing – original draft. **Liliana German‐Castelan:** Writing – original draft. **Vanesa S. Mattera:** Writing – original draft. **Pedzisai Mazengenya:** Writing – original draft. **Cecilia Skoug:** Writing – original draft. **Hisashi Umemori:** Conceptualization; writing – original draft; supervision; writing – review and editing.

## CONFLICT OF INTEREST STATEMENT

The authors declare no conflicts of interest.

### PEER REVIEW

The peer review history for this article is available at https://www.webofscience.com/api/gateway/wos/peer‐review/10.1111/jnc.16258.

## Data Availability

Data sharing is not applicable to this article as no new data were created or analyzed in this study.
